# Homozygous variant p. Arg90His in *NCF1* is associated with early-onset Interferonopathy: a case report

**DOI:** 10.1186/s12969-021-00536-y

**Published:** 2021-04-23

**Authors:** Oskar Schnappauf, Liane Heale, Dilan Dissanayake, Wanxia L. Tsai, Massimo Gadina, Thomas L. Leto, Daniel L. Kastner, Harry L. Malech, Douglas B. Kuhns, Ivona Aksentijevich, Ronald M. Laxer

**Affiliations:** 1 National Human Genome Research Institute, National Institutes of Health, Bethesda, USA; 2 The Hospital for Sick Children, University of Toronto, Toronto, Canada; 3 National Institute of Arthritis and Musculoskeletal and Skin Diseases, National Institutes of Health, Bethesda, USA; 4 National Institute of Allergy and Infectious Diseases, National Institutes of Health, Bethesda, USA; 5 Frederick National Laboratory for Cancer Research, Frederick, USA

**Keywords:** Autoinflammation, Autoimmunity, Interferons, Systemic lupus erythematosus, NCF1

## Abstract

**Background:**

Biallelic loss-of-function variants in *NCF1* lead to reactive oxygen species deficiency and chronic granulomatous disease (CGD). Heterozygosity for the p.Arg90His variant in *NCF1* has been associated with susceptibility to systemic lupus erythematosus, rheumatoid arthritis, and Sjögren’s syndrome in adult patients. This study demonstrates the association of the homozygous p.Arg90His variant with interferonopathy with features of autoinflammation and autoimmunity in a pediatric patient.

**Case presentation:**

A 5-year old female of Indian ancestry with early-onset recurrent fever and headache, and persistently elevated antinuclear, anti-Ro, and anti-La antibodies was found to carry the homozygous p.Arg90His variant in *NCF1* through exome sequencing. Her unaffected parents and three other siblings were carriers for the mutant allele. Because the presence of two *NCF1* pseudogenes, this variant was confirmed by independent genotyping methods. Her intracellular neutrophil oxidative burst and *NCF1* expression levels were normal, and no clinical features of CGD were apparent. Gene expression analysis in peripheral blood detected an interferon gene expression signature, which was further supported by cytokine analyses of supernatants of cultured patient’s cells. These findings suggested that her inflammatory disease is at least in part mediated by type I interferons. While her fever episodes responded well to systemic steroids, treatment with the JAK inhibitor tofacitinib resulted in decreased serum ferritin levels and reduced frequency of fevers.

**Conclusion:**

Homozygosity for p.Arg90His in *NCF1* should be considered contributory in young patients with an atypical systemic inflammatory antecedent phenotype that may evolve into autoimmunity later in life. The complex genomic organization of *NCF1* poses a difficulty for high-throughput genotyping techniques and variants in this gene should be carefully evaluated when using the next generation and Sanger sequencing technologies. The p.Arg90His variant is found at a variable allele frequency in different populations, and is higher in people of South East Asian ancestry. In complex genetic diseases such as SLE, other rare and common susceptibility alleles might be necessary for the full disease expressivity.

**Supplementary Information:**

The online version contains supplementary material available at 10.1186/s12969-021-00536-y.

## Background

NCF1 (p47^phox^) is a component of the phagocytic NADPH oxidase complex type 2 (NOX2) that upon sensing of pathogenic stimuli releases reactive oxygen species (ROS) into phagosomes and the extracellular compartment. During this process, cytosolic NCF1 gets phosphorylated and interacts with NCF2 and NCF4. This ternary structure gets translocated to the plasma membrane where it associates with the cytochrome complexes CYBB and CYBA to form NOX2. Subsequently, the NOX2 complex transports electrons from NADPH to oxygen, resulting in the release of a variety of ROS. Loss-of-function variants in *NCF1* and other genes coding for components of the NOX2 complex are associated with chronic granulomatous disease (CGD), a primary immunodeficiency that is characterized by granulomatous inflammation and recurrent infections due to defects in ROS-dependent destruction of phagocytized microorganisms. The rare missense variant p.Arg90His (rs 201802880, gnomAD MAF = 0.007) in *NCF1* was reported as a complex-disease susceptibility factor for systemic lupus erythematosus (SLE) and other autoimmune diseases [1, 2]. In these studies, the p.Arg90His variant was associated with impaired extracellular ROS production and hyperactivation of the interferon (IFN) type 1 signaling but not with a full CGD clinical phenotype. Aside from its role in phagosome-mediated pathogen clearance, ROS also exhibit intra- and intercellular signaling properties and play an important role in the regulation of inflammation and immune responses [3–5]. Interferon (IFN) signaling is the main mediator of antimicrobial mechanisms and recent studies have suggested that neutrophil-derived ROS suppress the activity of type I IFN that is produced by plasmacytoid dendritic cells (pDCs) [6]. pDCs are a unique subset of dendritic cells and the main producers of IFN cytokines in patients with SLE [7]. pDC-mediated IFN induces IL-15 production by conventional DCs (cDCs) which in turn activates IFN type II signaling in natural killer cells [8].

Here we characterize a female patient, homozygous for p.Arg90His in *NCF1*, who presented with autoinflammatory and autoimmune features accompanied by a strong upregulation of IFN-regulated genes. Overall, her clinical features were most consistent with a periodic fever syndrome, while her laboratory findings were suggestive of an autoimmune disorder.

## Case presentation

**Fig. 1:**
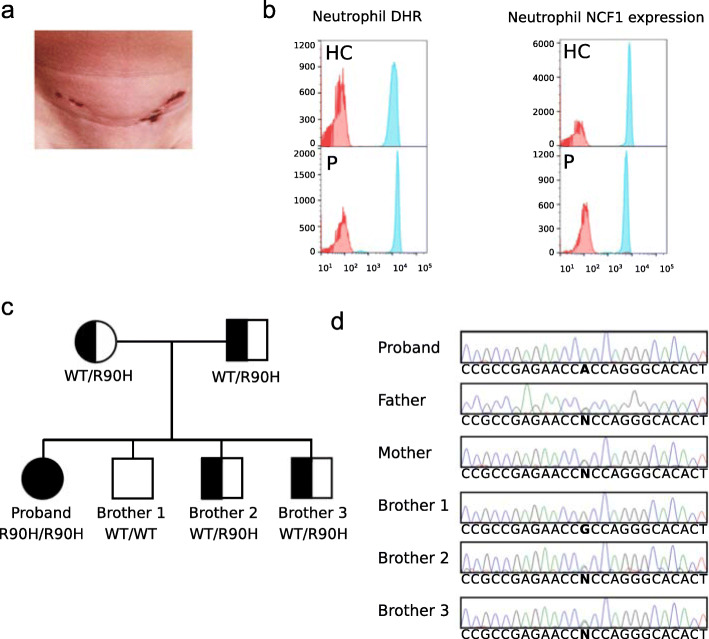
Patient presentation and *NCF1* p.Arg90His genotyping in the proband and her family. **a**: Atrophic skin lesion on lower abdomen of patient homozygous for *NCF1*, p.Arg90His. **b**: Residual oxidase activity of neutrophils and NCF1 expression in patient and healthy control. Left: Residual oxidase activity of neutrophils was determined using dihydrorhodamine (DHR) oxidation by flow cytometry. Right: NCF1 expression in patient and healthy control. NCF1 expression in neutrophils is presented as mean fluorescence intensity. Both panels: Red histograms represent neutrophils treated with buffer under basal conditions; blue histograms represent neutrophils in response to PMA (400 ng/mL). **c**: Pedigree of the family with the recessively inherited homozygous pathogenic variant p.Arg90His in the *NCF1* gene. **d**: Sanger sequencing validation in proband and family. Sanger sequencing confirmed the homozygous variant *NCF1*, c.269G > A, p.Arg90His in the patient. Her parents and two brothers are heterozygous for the same variant

The patient was born at 39 weeks of gestation with a birth weight of 2.7 kg and normal Apgar score. At age 18 months, she developed episodes of fever (up to 104.0 °F), anorexia and lethargy that recurred every 6–8 weeks lasting for 7–10 days. Two years into the course of her illness, she began experiencing nausea, vomiting and severe headache with each fever episode. During one episode, her cerebrospinal fluid and brain MRI findings were consistent with aseptic meningitis. She showed signs of failure to thrive, iron deficiency anemia, atrophic skin lesions (Fig. 1a).

Autoantibody testing revealed positive ANA (1:640), anti-Ro (> 100 U/mL) and anti-La (> 12 U/mL) antibodies. The remainder of her specific autoantibodies, including anti-dsDNA and rheumatoid factor, were negative. Both during and between fever episodes, the patient had marked elevation of the erythrocyte sedimentation rate (ESR), but normal to only mildly elevated levels of C-reactive protein (CRP). With two of the fever episodes, she developed a mild macrophage activation syndrome (MAS) with raised serum ferritin, neutropenia, and thrombocytopenia. Otherwise, her complete blood count was normal. Abdominal ultrasound identified small lymph nodes in peripancreatic and splenic hilum regions and chest x-ray showed mild bilateral perihilar peribronchovascular linear opacities. The Schirmer test for ocular dryness and Rose Bengal ocular staining did not show any ocular sicca. She had no clinical stigmata of SLE, Sjögren syndrome or other autoimmune disease until the age of seven, when she developed the first of two episodes of parotitis that resolved spontaneously (Table 1). A complete set of investigations for recurrent fever did not reveal any infectious or malignant etiology. Due to the absence of infections and a normal neutrophil oxidative burst capacity, her clinical features were not consistent with typical CGD (Fig. 1b).

**Table 1:** Comparison of clinical features of patient with homozygous *NCF1*, p.Arg90His to pediatric SLE patients

Disease Features	Pediatric SLE Patients^a^	***NCF1*** Variant Patient
Age of disease onset	Average 12 years	18 months
Fever	35–100%	+
Pattern	With active disease	Recurrent episodes
Cutaneous Involvement	60–90%	+
Manifestations	Malar rash; photosensitivity; discoid rash; mucosal ulceration	Inflamed linear lesion with atrophic scar
Alopecia	10–30%	+
Arthritis	60–90%	–
Neuropsychiatric involvement	15–95%	+
Manifestations	Headaches; cognitive dysfunction; seizures; psychosis	Severe headache with fever
Pericarditis	20–30%	–
Pleuritis	20–30%	–
Renal Disease	48–100%	–
Gastrointestinal Disease	24–40%	+
Manifestations	Peritonitis (sterile); abnormal liver function; pancreatitis; colitis	Focal minimal triaditis
Hematological disorders	33–75%	+
Manifestations	Anemia; lymphopenia > neutropenia; thrombocytopenia	Chronic anemia; intermittent thrombocytopenia, neutropenia and lymphopenia
Inflammatory Markers	ESR correlates with active disease; CRP often normal	ESR elevated disproportionate to CRP with fever
Autoantibodies		
ANA	> 99%	+
Anti-ds DNA	84–100%	–
Anti-Sm	23–48%	–
Anti-Ro	38–54%	+
Anti-La	16–32%	+

*SLE* systemic lupus erythematosus; *ESR* erythrocyte sedimentation rate; *CRP* C-reactive protein; *ANA* antinuclear antibody; *ds* double-stranded; *Sm* Smith

^a^Adapted from Cassidy JT, Petty RE, Laxer RM, Lindsley CB. Textbook of Pediatric Rheumatology, 6th edition. 2011. Saunders Elsevier; Philadelphia, PA

**Fig. 2:**
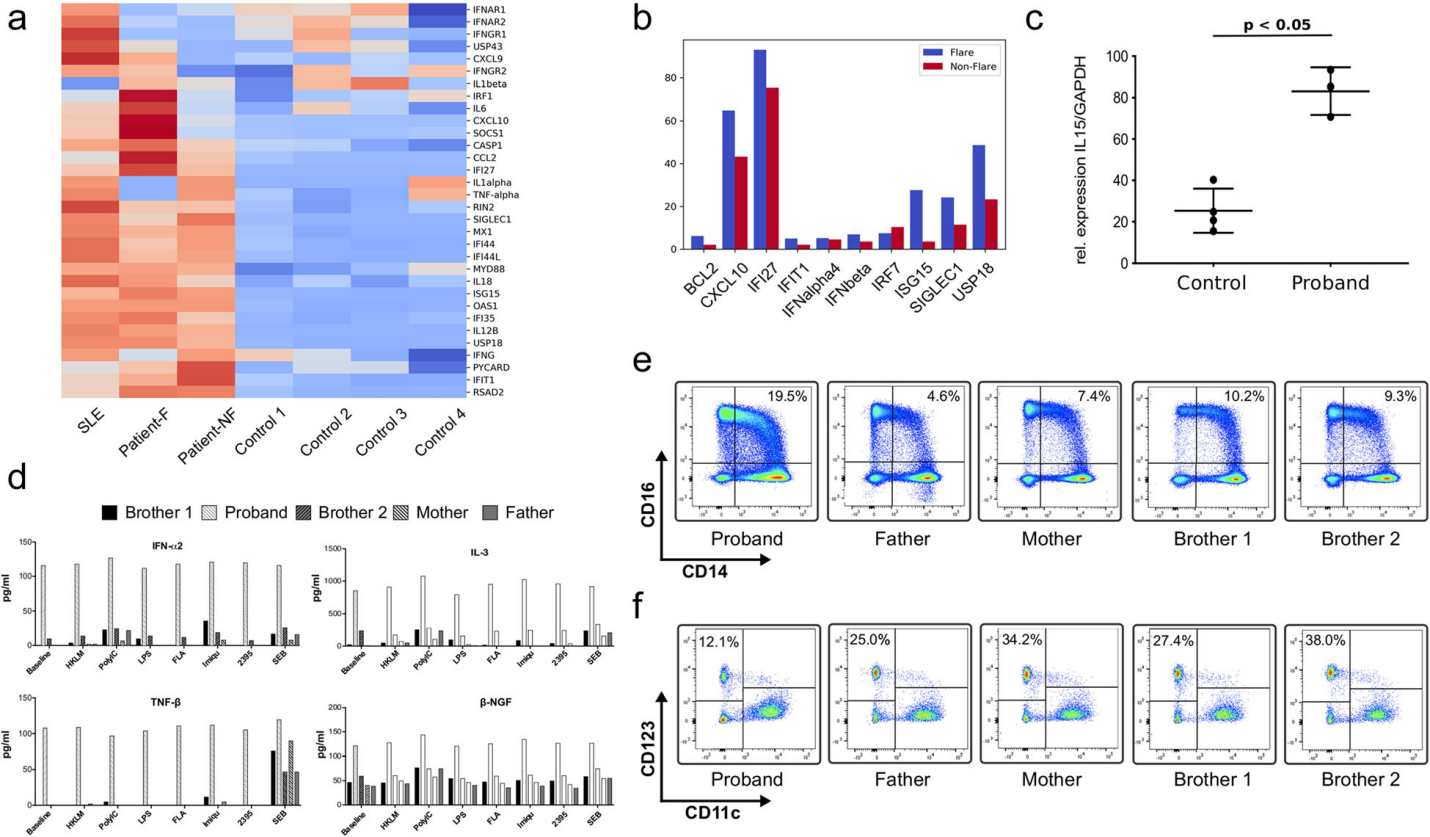
Interferon signature analysis, inflammatory cytokine profile and immune phenotyping in patient and family. **a**: Nanostring analysis for IFN signature genes in the patient (Patient-F = sample taken during flare; Patient-NF, SLE patient with the complement C1R deficiency (SLE) and healthy controls (Control 1–4). Red, elevated expression compared to control; blue, reduced expression compared to control. **b**: Quantitative reverse transcription (qRT-PCR) analysis of 10 IFN-related genes shows strong upregulation in the proband compared to control. Shown is fold change to control normalized to *GAPDH* expression. **c**: Quantitative reverse transcription (qRT-PCR) analysis of IL15 in proband and healthy controls. Shown are the relative expression values of *IL15*/*GAPDH*. **d**: IFN-α2, IL-3, TNF-β and nerve growth factor β (NGFβ) cytokine measurement on whole blood cells from the proband, her parents and 2 brothers. Cells were untreated or stimulated with different stimuli (heat-killed *Listeria monocytogenes* [HKLM] at 107 bacteria/ml, Poly(I:C) at 10 μg/ml, lipopolysaccharide [LPS] at 1 μg/ml, flagellin [FLA] at 50 ng/ml, imiquimod [Imiqu] at 5 μg/ml, ODN2395 [2395] at 5 μM and Staphylococcal Enterotoxin B [SEB] at 1 μg/ml). **e**: Quantification of pDCs and intermediate monocytes in the proband, her parents and two brothers. PBMCs were extracted from whole blood and incubated with the monoclonal antibodies anti-CD11c and anti-CD123 for pDC analysis and anti-CD14 and anti-CD16 for the analysis of intermediate monocytes. The data shown here are the only comparisons that achieved nominal statistical significance by the Mann-Whitney U test, which would not withstand correction for multiple comparisons

Her fever episodes responded well to systemic steroids (Dexamethasone, 0,25–4.5 mg) and recurred upon weaning. A trial of hydroxychloroquine did not alter the frequency or severity of disease flares. Given features of MAS with her febrile episodes, and the responsiveness of MAS in other situations to IL-1 inhibition, she received treatment with anakinra (100 mg [6 mg/kg]), at the onset of the fever, which reduced the height of the fever peaks to some extent but did not completely abort the episodes. An attempt at daily prophylactic anakinra also did not reduce the frequency of episodes, suggesting that her disease was not primarily mediated by dysregulated interleukin-1 activity. In view of results that showed upregulation of predominantly interferon-stimulated genes (ISGs) (see below), our patient was treated with the JAK inhibitor tofacitinib (5–10 mg), which resulted in decreased serum ferritin levels and frequency of fevers (Suppl. Fig. 1C), but only a partial clinical effect was seen. Our patient was subsequently started on sirolimus (rapamycin, 2 mg), with which we have been able to wean off her corticosteroids while maintaining complete resolution of fevers. Exome sequencing (ES) was performed on the patient and her parents, who are of Indian ancestry. The proband was found to be homozygous for the rare missense variant p.Arg90His in the *NCF1* gene, while her parents were healthy carriers for this variant (Fig. 1c). Three other healthy siblings were either carriers for the variant or wildtype. No other plausible candidate gene variants were identified under the assumption of either a dominantly or a recessively inherited disease (Suppl. Table 1). Since the presence of two pseudogenes, *NCF1B/C*, might interfere with the alignment algorithms, the GTGT sequence at the start of exon 2 of *NCF1* was used to discriminate between *NCF1B/C* and *NCF1* [1]. This genotype was confirmed by Sanger sequencing in the patient and her family members (Fig. 1d and Suppl. Fig. 1A). To determine the copy number of *NCF1B/C* and *NCF1*, a ddPCR assay containing probes specific for either the GTGT in *NCF1* or the ΔGT sequence in *NCF1B/C* was performed [9]. Since a total of 6 copies of *NCF1/NCF1B/NCF1C* are expected, healthy controls are predicted to have 2 GTGT copies vs 4 ΔGT copies expressed as 2/6. In p47phox CGD, the most common pathogenic variant is ΔGT in *NCF1*. Individuals who are carriers for this CGD-associated p47phox variant are predicted to have 1 GTGT copy and 5 ΔGT copies, or 1/6. In contrast, p47phox CGD patients are predicted to have 0 GTGT copies and 6 ΔGT copies, or 0/6. The proportional ratio of GTGT/(GTGT+ΔGT) of the patient and the healthy control sample were 2/6 which is equivalent to the expected 2 GTGT copies and 4 ΔGT copies (Suppl. Fig. 1B). A custom designed Nanostring-RNA expression array of 32 IFN-regulated and other inflammatory genes showed moderate to strong upregulation of predominantly interferon-stimulated genes (ISGs) in peripheral blood of the patient during (Patient-F) and in between flares (Patient-NF) compared to heathy controls. A patient with SLE due to complement C1R deficiency served as a positive control (Fig. 2a) [10]. Quantitative RT-PCR for 10 IFN-induced genes confirmed the Nanostring-RNA analysis. The strongest upregulation during and in between flares was seen for *IFI27*, *CXCL10*, *USP18,* and *ISG15* (Fig. 2b). Of note is that the expression of receptors for type I and type II IFN signaling (IFNAR1, IFNAR2, and IFNGR) was mostly downregulated in the patient, which raises the question whether type III IFN signaling pathway may be contributory to the interferon signature. Additionally, by qRT-PCR we showed that RNA expression of the type I IFN-induced cytokine IL-15 was significantly elevated in the patient (*p*-value = 0.001; Fig. 2c). Together these data corroborate that the enhanced inflammatory phenotype in this patient is mediated by an upregulation in interferon signaling pathways.

Whole blood cell cytokine analysis, either unstimulated or stimulated, showed strong differences in the patient’s cytokine profile compared to healthy family members and confirmed the observed type I IFN gene expression signature. The most elevated cytokines at baseline were IFN-α2, IL-3, TNF-β and β-NGF. All four cytokines were not further upregulated upon various stimulations (Fig. 2d). Interestingly, elevated serum concentrations of three of the four upregulated cytokines were previously associated with increased disease activity and clinical severity of SLE [11–14].

Flow cytometry immunophenotyping analysis in the patient revealed an increase in intermediate monocytes (CD14^++^CD16^+^) compared to her healthy family members (Fig. 2e). Intermediate monocytes play an important role in disease progression and severity in SLE, rheumatoid arthritis and autoinflammatory diseases [15–17]. Furthermore, a reduction in the patient’s pDCs (CD123^+^/CD11c^−^) was observed (Fig. 2f). This finding is in agreement with previous reports on reduced numbers of pDCs in peripheral blood in patients with SLE and type I interferonopathies [18]. Early studies suggested that reduced levels of peripheral pDCs were due to their localization in specific tissues and consequently, high numbers of pDCs were identified in the skin and kidneys of patients with autoimmune disease [19]. Subsequently these studies concluded that active pDCs have migrated to the sites of inflammation [20]. Activated pDCs express high levels of C-C-Motiv-Chemokine-Receptor (CCR) 5 and CCR7 [21] which are responsible for pDC-migration to lymphoid organs and inflamed organs [22]. Interestingly, later studies report normal to increased pDC numbers in the periphery of SLE patients [23, 24]. These distinct observations are likely due to differences in disease state and progression and it is likely that pDCs that were recruited to specific target organs display different characteristics than peripheral blood pDCs.

## Discussion and conclusions

In 2017, the rare variant p.Arg90His in *NCF1* was associated with susceptibility to autoimmune diseases in various populations and a significantly younger age of diagnosis of SLE (30.3 vs. 35.9 years; *p* = 2.0 × 10–6) [1, 2]. Individuals who carry the p.Arg90His variant do not present with immunodeficiency, indicating a sustained ability to generate a phagosomal respiratory burst. The phox homology (PX) domain of NCF1 exhibits a strong binding affinity to the plasma membrane component phosphatidylinositol-3,4-bisphosphate, while NCF4 preferentially binds to phosphatidylinositol-3-phosphate, highly abundant in the phagosomal membrane [25]. The p.Arg90 variant is located in the phosphoinositide-binding pocket of the PX domain of NCF1 and mutagenesis of this residue mainly reduces binding of cytosolic NCF1 to the plasma membrane but has minimal effect on translocation to the phagosomal membrane [26]. Patient cells carrying the p.Arg90His variant exhibit normal intracellular levels of ROS but show reduced extracellular ROS production in neutrophils [2]. In agreement with this, our patient exhibits a normal intracellular neutrophil oxidative burst capacity in response to PMA and has not had any significant infections. Reduced neutrophilic ROS release can trigger IFN gene expression by upregulation of IL-15 signaling, and elevated IL-15 levels induce exaggerated autoantibody production through activation of IFN-γ in NK cells [27]. In line with this, our patient shows high IL-15 cytokine gene expression levels and upregulation of IFN-regulated genes. Interestingly, CGD patients have increased risk of developing autoimmune disorders and were shown to exhibit increased expression of IFN-regulated genes [28, 29].

Olsson et al. demonstrated an association of p.Arg90His with IFN type I signaling in a cohort of patients with rheumatoid arthritis but not SLE. They speculated that extracellular ROS is important for the initiation of IFN type I signaling, but once initiated, IFN signaling is self-sustaining [2]. Thus, the strong IFN signature in fully developed SLE may outweigh the genetic effect of the p.Arg90His variant.

Our patient exhibits persistently high titer autoantibodies, including ANA, anti-Ro and anti-La, which are commonly seen in SLE or primary Sjögren syndrome. Despite the biological markers, she showed no clinical features of either disease until the age of seven, when she developed her first episode of recurrent parotitis. This is in agreement with the observation that onset of clinical SLE is preceded by the development of a variety of autoantibodies many years before the first clinical signs of disease [30]. Similarly, as seen in patients with SLE and not in patients with autoinflammatory diseases associated with inflammasome activation, our patient had a consistently normal or only minimally elevated CRP and a poor response to IL-1 blockade.

The finding of an ISG signature in our patient suggested that blocking interferon signaling may be an effective treatment for her disease. Type I interferon receptors signal via Janus kinase (JAK) 1 and Tyrosine Kinase 2 (TYK2), while Type II interferon receptor ligation results in JAK1 and JAK2 activation [31]. Our patient was treated with the JAK inhibitor tofacitinib and while this confirmed a partial effect, we speculate that the lack of full response may relate to the specificity of kinase inhibition by tofacitinib, as it has been shown to act on JAK3 more effectively than JAK1, JAK2 or TYK2 [32]. As we were unable to obtain an alternate JAK inhibitor for the patient, she was started on a trial of sirolimus (rapamycin), with which we have been able to wean off her corticosteroids while maintaining complete resolution of fevers. Of note, sirolimus has previously been shown to act on pDCs by suppressing their production of type I interferons [33].

Due to the presence of two highly homologous, non-functional *NCF1* pseudogenes, the p.Arg90His variant was not previously identified in GWAS as a susceptibility allele for SLE and correct genotyping of the *NCF1* gene requires highly specific methods, including exact copy number determination and *NCF1*-specific PCR techniques. It is also important to note that allele frequency databases such as the Genome Aggregation Database (gnomAD) and the 1000 Genomes Project use short sequence reads and might therefore fail to correctly identify the p.Arg90His and other variants in this gene.

The allele frequency of p.Arg90His variant differs in various populations, with highest numbers in East Asians. This variant is far less common in European and South Asian, including Indian, populations [1, 34]. Because SLE is a polygenic disease, we considered a possibility that the early-onset severe symptoms in our patient might be explained by the presence of additional susceptibility alleles that cumulatively contribute to disease manifestations. Such synergistic interactions between susceptibility alleles are a well-characterized disease mechanism in SLE and many other autoimmune diseases [35, 36]. Risk loci not only can affect age of onset, but also are strongly associated with a severity of clinical manifestations including immunological and hematologic disorder, renal disease, and mucocutaneous ulceration [36]. We therefore determined the presence of common and rare SLE-associated variants in exome data from this family and did indeed find an enrichment of other risk alleles in our patient (Suppl. Table 2). Compared to the other 3 siblings, the patient inherited more risk variants either in a heterozygous or homozygous state. The distribution of these variants was most similar with Brother 1, yet the patient carries additional risk alleles at two gene loci (*DNASE1* and *TYK2*). The additive effect of these SLE-associated variants may further contribute to the severity and earlier onset of disease in our patient.

In summary, the p.Arg90His variant was reported previously as a susceptibility allele in adults with a fully developed autoimmune phenotype. The present work provides evidence that homozygosity of this variant can be associated with childhood-onset immune dysregulation that includes features of systemic inflammation, including dysregulated interferon activity, and persistently elevated autoantibodies. This finding is of particular interest since our patient may be evolving toward a fully developed autoimmune phenotype later in life. Furthermore, these findings suggest that patients with unexplained recurrent fever and autoantibodies may have a genetic disorder in the interferon signaling pathway that should be investigated by interferon gene signature testing and / or genetic testing. Such discoveries might help in the diagnosis of other patients with atypical manifestations of SLE and autoinflammatory disease and also guide new targeted therapies.

## Supplementary Information

**Additional file 1.**

## Data Availability

All data generated or analyzed during this study are included in this published article. Exome sequencing raw data is available upon request.

## Abbreviations

cDCs: Conventional DCs
CGD: Chronic granulomatous disease
CRP: C-reactive protein
ESR: Erythrocyte sedimentation rate
IG: Immunoglobulin
ISG: Interferon-stimulated genes
JAK: Janus kinase
MAS: Macrophage activation syndrome
NOX2: NADPH oxidase complex type 2
PX: Phox homology
pDCs: Plasmacytoid dendritic cells
ROS: Reactive oxygen species
SLE: Systemic lupus erythematosus
TYK: Tyrosine Kinase
