# Supplementary material for: Homozygous variant p. Arg90His in NCF1 is associated with early-onset Interferonopathy: a case report
Source: Pediatr Rheumatol Online J. 2021 Apr 23;19:54. doi: 10.1186/s12969-021-00536-y (PMC8063424; doi:10.1186/s12969-021-00536-y)
Supplement: Supplementary file 1 — Additional file 1. [file 12969_2021_536_MOESM1_ESM.docx]

Supplementary Data

# Supplementary Methods and Materials

## Patient

The patient was followed at The Hospital for Sick Children in Toronto, Canada. She was also evaluated at the National Institutes of Health Clinical Center, Bethesda, Maryland. The patient and her healthy family members provided signed informed consent to participate in the study and to publish the material. The study was approved by the NIDDK/NIAMS Institutional Review Board (14-AR-0200).

## Exome sequencing

Genomic DNA samples for whole exome sequencing were extracted from peripheral blood using the Maxwell® 16 Blood DNA Purification Kit (Promega). Exome sequence libraries were prepared using an Illumina TruSeq DNA Sample Preparation Kit version 2 and paired-end sequencing was performed on an Illumina HiSeq2000 instrument. For data analysis, the GATK base quality score recalibration, indel realignment, duplicate removal, SNP/INDEL discovery and genotyping across all 6 samples (patient and 5 healthy family members) was performed simultaneously using standard hard filtering parameters according to GATK Best Practices recommendations. Gemini and exomiser software packages were used to further prioritize the variants (10, 11).

## NCF1 copy number determination and sequencing

## The number of NCF1 and NCF1B/C copies in the genome of the patient and healthy controls was determined using a droplet digital PCR with two distinct probes, recognizing either wild-type NCF1 sequence or the pseudogenes (12). For the confirmatory sequencing of the p.Arg90His variant, NCF1-specific primers were used as described previously (1) and shown in figure 1B.

## Nanostring analysis and real-time quantitative reverse transcription PCR

Whole blood RNA was extracted using a PAXgene Blood RNA system (Qiagen). Gene expression analysis was performed on 32 IFN-regulated genes using the nCounter Analysis System (NanoString Technologies). Total RNA (100 ng) was mixed with capture and reporter probes and hybridized on an nCounter Prep Station. For data analysis, nSolver and MatLab software was used. The expression data were normalized to the geometric mean of results obtained with housekeeping genes. For qRT-PCR, 250ng of total RNA was reverse transcribed using SuperScript™III Reverse Transcriptase (Thermo Fisher Scientific), and qRT-PCR was performed using PowerUP SYBR Green (Thermo Fisher Scientific). Probe-Primer-Mix was purchased from Thermo Fisher Scientific. Samples were run in duplicate on a ViiATM 7 Real-Time PCR System, and data were analyzed using the same system (Applied Biosystems).

## Whole blood cell stimulation and multiplex immunoassays of human cytokines

Whole blood samples from the patients and healthy family members were collected in sodium heparin BD Vacutainer tubes (Becton Dickinson). The samples were diluted 1:2 with RPMI 1640 medium (Gibco), and then stimulated with different stimuli: heat-killed Listeria monocytogenes (HKLM) at 107 bacteria/ml, Poly(I:C) at 20 μg/ml, lipopolysaccharide (LPS) at 1 μg/ml, flagellin at 250 ng/ml, imiquimod at 5 μg/ml, ODN2395 at 3 μg/ml, and staphylococcal enterotoxin B (SEB) at 0.4 μg/ml. The cells were incubated at 37°C, 5% CO2 for 22 hours. The supernatants were then collected after centrifugation at 1000g for 10 minutes and stored at -80° C. Cytokine levels from stimulated and unstimulated whole blood cells were detected using the Bio-Plex pro human cytokine 27-plex and 21-plex immunoassays (Bio-Rad) according to the manufacturer’s instructions. Each sample was assessed in duplicate. The concentrations of the 48 cytokines were determined according to the assay standard curve.

## Flow cytometry immunophenotyping

The red blood cells from whole blood samples were lysed with pharm lysis buffer (BD, 1:10 ratio) for 10 min at room temperature. The samples were centrifuged at 600 x g for 8 min. The cellular pellets were suspended and washed twice with FACS buffer (0.5% bovine serum albumin, 2 mM EDTA, 0.1 % sodium azide in phosphate-buffered saline). After these washing steps, the pellets were resuspended in FACS buffer and divided into separated tubes for cell surface staining. A set of antibodies to surface markers were added to the tubes. For the Monocyte/Dendritic cell/Natural killer cells: anti-human CD3, CD19, CD20, CD16, CD11c, CD56, CD123, CD14 and HLA-DR. For the B cells: anti-human CD3, CD19, CD20, CD24, CD27, CD38 and IgD. The cells were stained for 1 hour at 4° C. At least three million cells/events were collected for each cell types using a BD LSR Fortessa cytometer. Single color compensation beads were used to compensate the overlap of nine fluorophores. Flowjo 7.6 software was used to analyze the flow data.

## Analysis of polymorphonuclear neutrophil H2O2 production and NCF1 expression

NADPH oxidase activity of individual cells was measured by flow cytometry using dihydrorhodamine 123 (DHR). NCF1 expression was determined by permeabilization and fixation of whole blood and staining with anti-NCF1 antibody (Abcam, Cambridge, US) as described previously (12).

## NCF1B-, NCF1C- and NCF1-copy number determination and NCF1-specific sequencing

To exclude the influence of NCF1B- and NCF1C-pseudogenes and to obtain the correct sequence for the NCF1 gene, NCF1-specific primers were used for PCR-amplification as described before (Tab. S1) [6]. Sanger sequencing was performed using the BigDye Terminator Cycle Sequencing kit on a 3130xl Genetic Analyzer (Applied Biosystems). M13 forward and reverse primer were used for the sequencing reaction. The analysis was done using the Sequencher software package (Gene Codes).

## Real-time quantitative reverse transcription PCR (qRT-PCR)

RNA was isolated using PAXgene Blood RNA system (Qiagen). 250ng of total RNA was reverse transcribed using SuperScript™III Reverse Transcriptase (Thermo Fisher Scientific), and qRT-PCR was performed using PowerUP SYBR Green (Thermo Fisher Scientific). Probe-Primer-Mix was purchased from Thermo Fisher Scientific. Samples were run in duplicate on a ViiATM 7 Real-Time PCR System, and data were analyzed using the same system (Applied Biosystems).

## NanoString Assay

We extracted RNA using a PAXgene Blood RNA system (Qiagen) and conducted gene expression analysis with the nCounter Analysis System (NanoString Technologies) using a code set designed to target 32 IFN induced genes. Total RNA (100 ng) was mixed with capture and reporter probes and hybridized on an nCounter Prep Station. For data analysis, nSolver software and MatLab software was used. The expression data were normalized to the geometric mean of results obtained with housekeeping genes.

# Supplementary Tables

**Supplementary Table 1.** Variants identified in either heterozygous (de novo) or homozygous state in the proband by exome sequencing.

| Chr | Pos. | Ref. | Var. | Type | Gene | Transcript and codon change | Amino acid change | MAF | Genotype Proband |
| --- | --- | --- | --- | --- | --- | --- | --- | --- | --- |
| 1 | 148594474 | C | T | SNV | NBPF15 | NM_001170755.2:c.1847C>T | Ser616Leu | 1.31E-03 | C/T |
| 1 | 12921332 | T | C | SNV | PRAMEF2 | NM_023014.1:c.1123T>C | Cys375Arg | N.A. | C/C |
| 2 | 201171014 | G | C | 5' UTR variant | SPATS2L | NM_001282744.1:c.-2782G>C | N/A | N.A. | C/C |
| 3 | 139106340 | T | C | SNV | SLC7A11 | NM_014331.3:c.850A>G | Thr284Ala | 1.83E-03 | C/C |
| 7 | **74193642** | **G** | **A** | **SNV** | **NCF1** | **NM_000265.5:c.269G>A** | **Arg90His** | **7.00E-03** | **A/A** |
| 9 | 138236305 | T | C | 3' UTR variant | C9orf62 | NM_173520.2:c.*46+6T>A | N/A | N.A. | C/C |
| 10 | 90986653 | T | G | Synonymous SNV | LIPA | NM_000235.3:c.537A>C | Ile179= | N.A. | T/G |
| 16 | 29888097 | G | C | SNV | SEZ6L2 | NM_001243332.1:c.2084C>G | Ala695Gly | 6.22E-06 | G/C |
| 17 | 6658969 | T | C | 5' UTR variant | XAF1 | NM_001353135.1:c.-39T>C | N/A | N.A. | C/C |
| 19 | 50412788 | C | T | SNV | NUP62 | NM_016553.4:c.277G>A | Gly93Ser | 2.02E-03 | T/T |
| 19 | 53393584 | A | G | 5' UTR variant | ZNF320 | NM_001351777.1:c.-73-3T>C | N/A | -1.00E+00 | G/G |
| 20 | 43043159 | G | A | SNV | HNF4A | NM_000457.3:c.505G>A | Val169Ile | 2.08E-03 | G/A |

# Chr, chromosome; Pos, position; Ref, reference sequences; Var, variation sequence; SNV, single nucleotide variant; UTR, untranslated region; MAF, minor allele frequency in the Genome Aggregation Database; N/A, not applicable

**Supplementary Table 2.** SLE susceptibility loci genotype in the proband and her family.

| **Chr** | **Pos** | **Gene** | **Consequence** | **gnomAD** | **Patient** | **Father** | **Mother** | **Brother 1** | **Brother 2** | **Brother 3** |
| --- | --- | --- | --- | --- | --- | --- | --- | --- | --- | --- |
| 1 | 12252954T>G | TNFRSF1B | c.587T>G:p.M196R | 0.223 | T**G** | TT | T**G** | T**G** | T**G** | T**G** |
| 1 | 161479744A>G | FCGR2A | c.500A>G:p.H167R | 0.479 | AA | AA | AA | AA | AA | AA |
| 3 | 58385094T>C | PXK | c.1172T>C:p.L391S | 0.0002761 | TT | TT | TT | TT | TT | TT |
| 4 | 102751075G>A | BANK1 | c.182G>A:p.R61H | 0.255 | **AA** | G**A** | G**A** | G**A** | **AA** | GG |
| 4 | 102839286G>A | BANK1 | c.1147G>A:p.A383T | 0.303 | G**A** | GG | G**A** | G**A** | G**A** | GG |
| 11 | 35243921G>A | CD44 | c.882-1G>A | 0.009578 | GG | GG | GG | GG | GG | GG |
| 11 | 71155071C>T | DHCR7 | c.289G>A:p.A97T | 1.90E-05 | CC | CC | CC | CC | CC | CC |
| 12 | 10532325T>C | KLRK1 | c.214A>G:p.T72A | 0.803 | **CC** | **CC** | **CC** | **CC** | **CC** | **CC** |
| 15 | 89450586G>T | MFGE8 | c.226C>A:p.L76M | 0.622 | **TT** | **TT** | G**T** | **TT** | G**T** | G**T** |
| 16 | 3707746G>A | DNASE1 | c.731G>A:p.R244Q | 0.371 | G**A** | GG | G**A** | GG | GG | G**A** |
| 16 | 31276810G>A | ITGAM | c.230G>A:p.R77H | 0.092 | GG | GG | GG | GG | GG | GG |
| 19 | 10472932A>G | TYK2 | NA | 0.508 | **GG** | **GG** | A**G** | A**G** | **GG** | A**G** |
| 19 | 10475651C>A | TYK2 | c.1084G>T:p.V362F | 0.275 | C**A** | CC | C**A** | CC | CC | C**A** |

# Risk alleles are highlighted in red. Chr, chromosome; Pos, position; SNP, single nucleotide polymorphism; gnomAD, minor allele frequency in the Genome Aggregation Database (gnomAD)

# Supplementary Figure 1

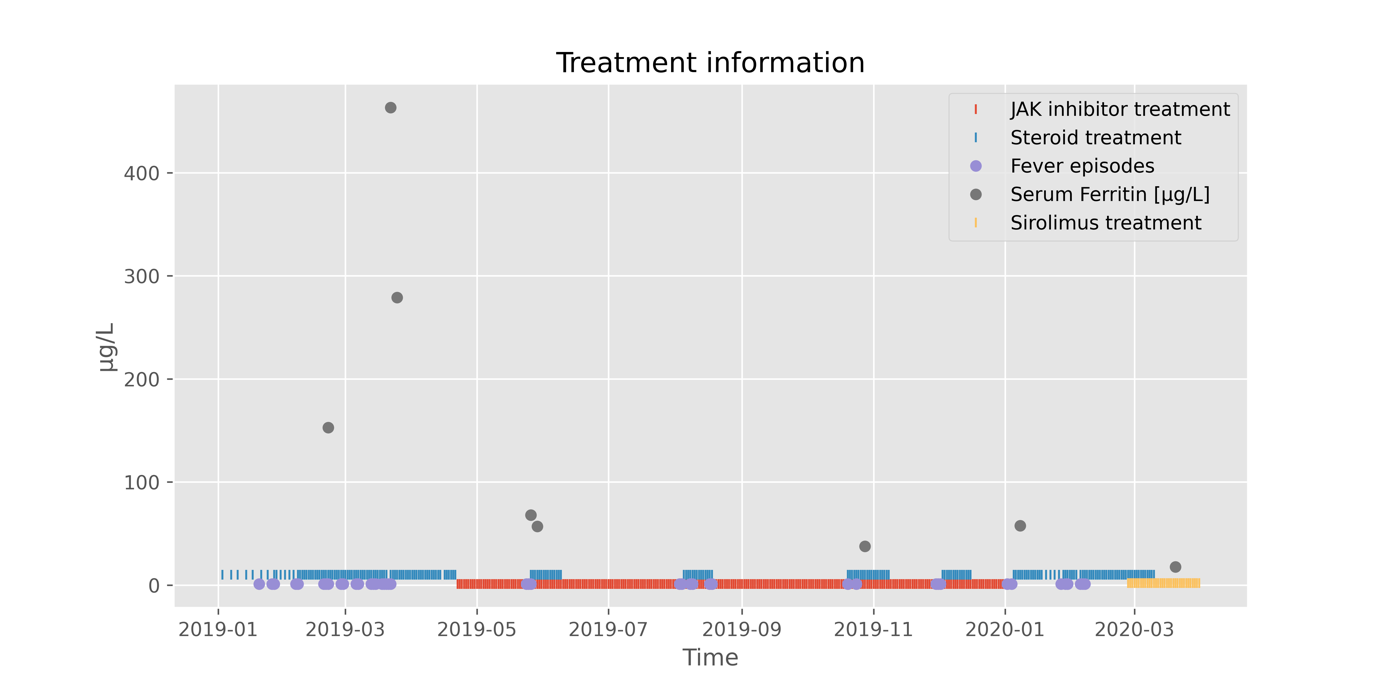


**Supplementary Figure 1.**

**A**: Top: Schematic representation of the *NCF1* gene and the NCF1 domain organization (phox homology [PX] domain, N-SH3 [SH3-1], C-SH3 [SH3-2], PBR/AIR [AIR] domain), showing the position of the p.Arg90His variant within the PX domain. Bottom: Schematic overview of the *NCF1*-specific PCR strategy. To prevent off-target amplification of *NCF1B* and *NCF1C*, *NCF1*-specific primer for the PCR amplification were used.

**B**: Digital droplet PCR analyses of *NCF1* in genomic DNA from proband, CGD patient, carrier and healthy control confirms the expected allele copy number ratio of *NCF1* to *NCF1B/C*.

**C**: JAK inhibitor, corticosteroid, and Sirolimus treatment from January 2019 to March 2020. Initiation of JAK inhibitor treatment (Tofacitinib, alternating 5 and 10 mg) in April 2019 reduced the frequency of the fever episodes and normalized serum ferritin levels in the patient. Subsequent Sirolimus (rapamycin, 2mg) treatment further helped to to wean off her corticosteroids and to reduce fever episodes.
